# Rrm3 and Pif1 division of labor during replication through leading and lagging strand G-quadruplex

**DOI:** 10.1093/nar/gkad1205

**Published:** 2023-12-20

**Authors:** Mor Varon, Daniel Dovrat, Jonathan Heuzé, Ioannis Tsirkas, Saurabh P Singh, Philippe Pasero, Roberto Galletto, Amir Aharoni

**Affiliations:** Department of Life Sciences and the National Institute for Biotechnology in the Negev, Ben-Gurion University of the Negev, Be’er Sheva 84105, Israel; Department of Life Sciences and the National Institute for Biotechnology in the Negev, Ben-Gurion University of the Negev, Be’er Sheva 84105, Israel; Institut de Génétique Humaine, CNRS, Université de Montpellier, Equipe Labellisée Ligue Contre le Cancer, 34396 Montpellier, France; Department of Life Sciences and the National Institute for Biotechnology in the Negev, Ben-Gurion University of the Negev, Be’er Sheva 84105, Israel; Department of Biochemistry and Molecular Biophysics, Washington University School of Medicine, St. Louis, MO 63110, USA; Institut de Génétique Humaine, CNRS, Université de Montpellier, Equipe Labellisée Ligue Contre le Cancer, 34396 Montpellier, France; Department of Biochemistry and Molecular Biophysics, Washington University School of Medicine, St. Louis, MO 63110, USA; Department of Life Sciences and the National Institute for Biotechnology in the Negev, Ben-Gurion University of the Negev, Be’er Sheva 84105, Israel

## Abstract

Members of the conserved Pif1 family of 5′-3′ DNA helicases can unwind G4s and mitigate their negative impact on genome stability. In *Saccharomyces cerevisiae*, two Pif1 family members, Pif1 and Rrm3, contribute to the suppression of genomic instability at diverse regions including telomeres, centromeres and tRNA genes. While Pif1 can resolve lagging strand G4s *in vivo*, little is known regarding Rrm3 function at G4s and its cooperation with Pif1 for G4 replication. Here, we monitored replication through G4 sequences in real time to show that Rrm3 is essential for efficient replisome progression through G4s located on the leading strand template, but not on the lagging strand. We found that Rrm3 importance for replication through G4s is dependent on its catalytic activity and its N-terminal unstructured region. Overall, we show that Rrm3 and Pif1 exhibit a division of labor that enables robust replication fork progression through leading and lagging strand G4s, respectively.

## Graphical Abstract

**Graphical Abstract:**
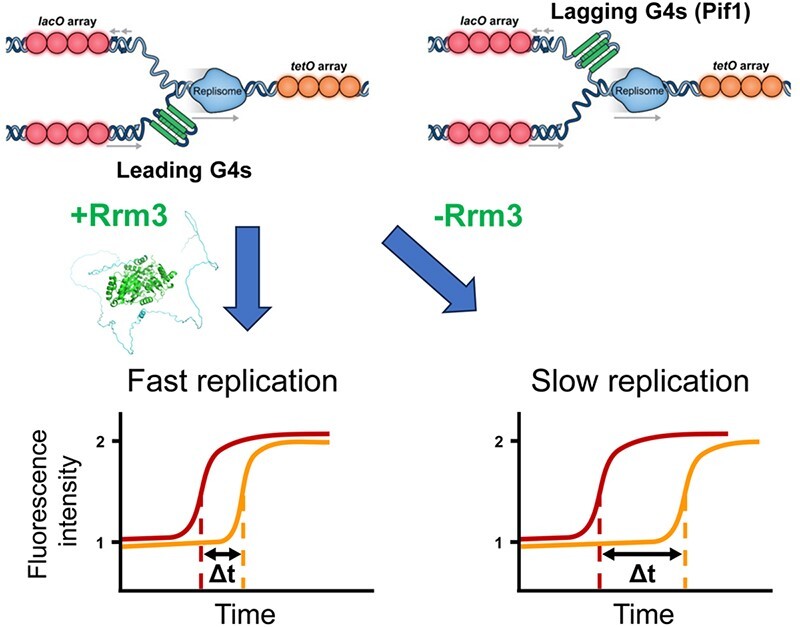

## Introduction

G-quadruplex structures (G4s) are stable non-canonical four-stranded DNA secondary structures formed by non-Watson–Crick base pairing (1). G4s are widely spread across prokaryotic and eukaryotic genomes, and many G4 motifs are evolutionarily conserved, suggesting that they have important roles in genome regulation (2,3). Formation of G4s can perturb DNA replication by acting as an obstacle to the progression of the replisome (4). Stalled replisomes at G4 sites can lead to increased levels of double strand breaks and recombination, thereby promoting genomic instability. Thus, mutations in DNA helicases that can unwind G4s, are linked to genomic instability and are associated with disease and cancer (5). While the relevance of G4s to genome stability is widely acknowledged, the factors and mechanisms by which cells mitigate their potential threat for genome stability remain unclear.

The Pif1 protein family, that includes highly conserved 5′-3′ DNA helicases, are found in all eukaryotes and in some prokaryotes. These helicases can process G4s and mitigate their negative impacts on genome stability (4). In *Saccharomyces cerevisiae*, two helicases of the Pif1 family, Pif1 and Rrm3, contribute to multiple DNA processes including telomere maintenance (6), centromere function (7), and Okazaki fragment maturation during DNA replication (8,9). Previous studies showed that Pif1 can efficiently unwind G4s *in vitro* and can also resolve G4s *in vivo* (10), thereby mitigating G4-induced mutagenesis and genome instability (11–13). However, Pif1 only enables replisome progression through G4s located on the lagging strand template (10), and it remains unknown whether similar mechanisms exist for replication through leading strand G4s. Rrm3 was shown to have a broad effect on genome maintenance including the suppression of recombination at repeat sequences (14) and prevention of replisome stalling at a variety of chromosomal loci including centromeres, telomeres, tRNA genes and transcribed genes (9). The ability of Rrm3 to prevent fork stalling at a variety of genomic locations has been associated with its ability to remove proteins bound to the DNA ahead of the replication fork (15). Yet, while Rrm3 is associated *in vivo* with many genomic processes overcoming various obstacles, less is known of Rrm3 activity at G4s and its importance for efficient replisome progression through G4 rich sequences.

Here, we have directly examined the importance of Rrm3 for DNA replication progression through G4 sequences. We have utilized our recently developed approach for measuring the progression rate of single replication forks (16), as they replicate through G4 sequences located on the leading or lagging strand template, in living cells (10). Using this approach, we found that Rrm3 is important for efficient replication through G4s located on the leading strand template, but not on the lagging strand template. We show that stabilization of leading strand G4s, by increasing the number of G4 repeats or by G4 stabilizing ligand, leads to increased levels of fork slowdown in the absence of Rrm3. Finally, we show that the importance of Rrm3 for replication through leading G4s is dependent on its catalytic activity and N-terminal unstructured region. Our results provide direct *in vivo* evidence for a function of Rrm3 at G4s and its importance for replication through G4 sequences within the leading strand.

## Materials and methods

### Strain generation

Strains for replication time measurement were generated on the background of a W303 MATa *Saccharomyces cerevisiae* strain, expressing LacI-HaloTag and tetR-tdTomato fusion proteins in the nucleus. *LacO*x256 and *tetO*x224 arrays are located at chrIV:332960 and chrIV:352560 respectively, near Autonomously Replicating Sequence (*ARS*) 413 with a mid-array distance of approximately 30.6 kb (16). *PIF1* was replaced by a *kanMX* antibiotic cassette using the Lithium Acetate (LiAc) transformation method. Rrm3-AID strain was generated by integrating a PCR cassette containing the auxin-inducible degron (AID) sequence followed by 6xFLAG-Tag and an *hphMX* antibiotic cassette at the 3′ end of the Rrm3 gene. The Oryza sativa *TIR1* gene was integrated at the Ade1 locus to facilitate Rrm3-AID degradation following auxin addition. Rrm3 truncation strains were generated on the background of Rrm3-AID strain by integrating the different truncations followed by 6xHA-tag into the *URA3* locus using an integrative plasmid. To generate the plasmids containing the Rrm3 truncations, a gBlock (IDT) containing the endogenous promoter of Rrm3 along with an NruI restriction site, 6xHA-tag and the *CYC1* terminator was inserted into the pRS306 integrative plasmid via Gibson assembly, following the manufacturer's protocol (NEB). Different truncations of the Rrm3 gene were then amplified from WT yeast genomic DNA and inserted into the plasmid that was restricted by NruI restriction enzyme via Gibson assembly (NEB). The G4 motifs were inserted 11.1 kb upstream of *ARS413* and 8.4 kb downstream of the *tetO* array. The Different G4 forming sequences were integrated into the *pif1*-deleted/Rrm3-AID cells by replacing a *natMX* cassette, inserted at the same genomic location, using a marker-free CRISPR/CAS9 approach as previously described (17). For the generation of a strain containing tRNA between the *lacO* and *tetO* arrays, the glutamate tRNA gene (YNCI0011W) was amplified from the yeast genome along with 362 bp of upstream and 214 bp of downstream sequences to maintain proper sequence context. The tRNA was inserted ∼3 kb downstream of the *lacO* array in a head-on (HO) orientation relative to the expected direction of replisome progression in this locus, by replacing the *natMX* cassette as described above. For the generation of strains for copy number variation analysis, G4_(A+B)_ or G4(6) were integrated into a *RRM3*-AID strain that does not contain the *lacO* and *tetO* arrays and the cognate repressors. These G4s were integrated at chrIV:332960, at a distance of 3 kb from *ARS413*. The G4_(A+B)_ sequence contains the G4_A_ motif, separated by 96 bp of a spacer sequence, followed by G4_B_ (10). The full sequence is GGGTACGGTGGGTAATAAGGGAAGGTATCGGGTTAGATCCCAGTCGAATGGATTAATCAAACAGATCTGTAGCCGGAGAGGCATACCCCCTGCGACACTTTACGAAGGCATCTGCAAAAATCATAACTGGGGAGGGGAAGGGGAGGGG where G4_A_ sequence is underlined first followed by an underline for G4_B_. The G4(4) and G4(6) sequences contain the core G4 motif (G_3_T_3_)_3_G_3_ (18), repeating 4 and 6 times, respectively, containing a random linker of 40 bp between each of the G4 motifs. These repeat G4 sequences were synthesized as synthetic genes (BioBasic). All replacements were validated with Polymerase Chain Reaction (PCR) followed by Sanger sequencing.

### Microscopy

Yeast cells were grown overnight in synthetic complete (SC) medium containing 4% glucose at 30°C. Yeast cultures were diluted at OD_600_ = 0.1 and SiR-HALO dye was added to a final concentration of 400 nM (19). One hour following SiR-HALO dye addition, 10 μg/ml of α-factor was added to arrest the cells in G1 phase, and the cultures were incubated for two additional hours to facilitate synchronization. In *RRM3*-AID strains, 1 mM of indole-3 acetic acid (IAA) was added 1 h prior to the experiment to facilitate the degradation of the endogenous Rrm3 protein. In experiments containing the PhenDC3 compound 10 μM of PhenDC3 were added immediately after the cells were diluted. Cells were then immobilized on microscopy slide chambers (Ibidi) coated with 2 mg/ml concanavalin A and washed thoroughly from α-factor and SiR-HALO dye with warm SC medium containing 4% glucose prior to microscopy experiments. Live-cell imaging of the cells was performed on an AxioObserver inverted wide-field microscope (Zeiss) with a Colibri 7 LED light source, at 1 min intervals for 4 h at 30°C, using a ×63 oil objective (NA = 1.4) in 3D (8 z-sections 0.8 μm apart). LacI-Halo-SiR and TetR-tdTomato were excited with 631 and 555 nm illumination, respectively.

### Copy number variation analysis

For copy number variation analysis, yeast cells were grown overnight at 25°C to a cell density of 5–6 × 10^6^ cells/ml in 200 ml of YPD medium. Cells were synchronized in G1 by the addition of 8 μg/ml of α-factor for 2:30 hours. Half an hour following α-factor addition, IAA was added to facilitate Rrm3 depletion only to the target cells at a final concentration of 1 mM. To slowdown replication forks, hydroxyurea (HU) was added 20 min prior to cell release to a final concentration of 50 mM. To release the cells into S-phase following α-factor synchronization, Pronase (Sigma) was added to a final concentration of 75 μg/ml. Immediately prior to cell release, 45 ml of culture was taken as a G1 control sample for the extraction of genomic DNA (gDNA). Following cell release into S-phase, 45 ml of culture samples were taken for gDNA extraction at 35, 47 and 60 min time points. In parallel, 0.5 ml of cell samples were taken from asynchronous population, G1 cells and 35, 47, 60 and 90 min following release into S-phase for flow cytometry analysis. For gDNA extraction, spheroplasts were prepared using Zymolyase 20T (Nacalai) according to standard protocol followed by lysis of the spheroplasts using G2 buffer (Qiagen) supplemented with RNaseA and proteinase K. Standard phenol–chloroform extraction was then used to separate the gDNA from the proteins and lipids followed by isopropanol DNA precipitation and the pellet was washed with cold 70% EtOH. The purity and concentration of all gDNA samples were verified by NanoDrop 1000 UV Visible Spectrophotometer (Thermo Fisher Scientific). DNA copy number levels at the vicinity of *ARS413* and *ARS607* were measured using real-time PCR (LightCycler 480 II, Roche) in 384-well plates using a series of primers located at the vicinity of these origins at a spacing of 1–2 kb distance (Supplementary Table S1). Primer specificity was first examined in silico by blasting primers sequences with the *S. cerevisiae* reference genome (*sacCer3*, April 2011 version). Only primers that hybridize once on the reference genome were considered as specific and were further tested for specificity and efficiency experimentally by qPCR using a range of genomic DNA concentrations (see below). The PCR program used for amplification included: 10 min at 95°C, then 45 cycles of: 95°C 10 s, 60°C 15 s and 72°C 15 s. Primer specificity and efficiency were determined using ‘Tm calling’ ‘Abs Quant:2^nd^ Derivative Max’ tools from the Light Cycler 480 software (Roche). All primers were designed to generate an amplicon of 80 bp and exhibited a single sharp peak in the melting curve analysis showing their high specificity. The primer efficiency calculation was performed by plotting the correlation of quantification cycle (Cq) to log DNA template concentrations (10, 1, 0.1 and 0.01 ng/μl) using the equation *y* = *Bx* + *A*. Primers efficiency was determined using the equation: *E* = 10^(–1/*B*)^. All the primers used in this study had an efficiency between 1.98 and 2.05 with a R fitting >0.999. The qPCR reactions (Roche LighCycler FastSTART DNA master SYBR green I) were performed according to the manufacturer instructions. Determination of DNA concentrations was performed by amplification of reference asynchronous gDNA at four different concentrations (10, 1, 0.1 and 0.01 ng/μl). Copy number levels at the vicinity of *ARS413* and *ARS607* were normalized to G1 levels (copy number of 1) to determine duplication level of replicated regions.

### Western blot analysis

For the detection of Rrm3-AID-6xFLAG by western blot, 50 ml of yeast cells were grown until late-exponential phase. Rrm3 depletion was induced by the addition of 1 mM of IAA for 1 h before harvest. Cells were centrifuged and pellets were frozen at −20°C overnight. Next, cell pellets were thawed and incubated in 1 ml of 0.2 M NaOH for 5 min, followed by boiling at 95°C for 5 min in 50 μl of Laemmli Buffer including 10% β-mercaptoethanol. Lysates were centrifuged for 2 min and 15 μl of the supernatants were loaded and run on a precast 10% SDS-PAGE gel (Bio-Rad). For the detection of Rrm3 different truncation fused to 6xHA, 2 ml of cells in OD_600_ = 1 were collected and 1 h after 1 mM IAA addition. Then, cells were centrifuged and pellets were resuspended in 500 μl ddH_2_O and 75 μl of 1.85 M NaOH/7.5% β-Mercaptoethanol for 15 min, followed by addition of 75 μl of 55% TCA solution. Cells were then centrifuged and the pellet was boiled at 95°C in loading buffer containing 8 M urea, 5% SDS, 200 mM Tris–HCl, 1 mM EDTA and 0.1% bromophenol blue. For each sample 10 μl of the supernatant was loaded and run on a precast 10% SDS-PAGE gel (Bio-Rad). Separated protein bands were transferred to a PVDF membrane using a Trans-Blot Turbo system (BioRad) followed by blocking with PBST (PBS buffer containing 0.1% Tween 20) + 5% skim milk for 1 h. The membrane was washed with PBST buffer 3 times for 5 min, and was incubated with a primary mouse anti-FLAG antibody conjugated with HRP enzyme (1:1000) (Sigma-Aldrich Cat# A8592) in PBST + 1% skim milk for 1 h. The membrane was washed three times and detected with the EZ-ECL Chemiluminescence detection kit (Biological Industries) according to the manufacturer's protocol. Detection of Rrm3 truncations were performed with anti-HA antibody (1:2000, Santa Cruz Cat# sc-7392) and a secondary goat-anti-mouse antibody (1:10 000) (Jackson Immunoresearch Cat# 115-035-003) conjugated with HRP. Detection of Pgk1 bands was performed with a primary mouse anti-Pgk1 antibody (1:5000, Invitrogen Cat# 459250) and a secondary goat-anti-mouse antibody (1:10 000, Jackson Immunoresearch Cat# 115=035-003) conjugated with HRP.

### Quantification and statistical analysis

Time-lapse measurements were collected with ZEN 3.0 and analyzed using a custom-made computational pipeline developed specifically for the analysis of replication rates (16). Our MATLAB-based pipeline identifies, tracks and quantifies the LacI-Halo-SiR and TetR-tdTomato dots in each cell. For each strain, at least 40 cells were measured in two independent experiments. Statistical analysis of replication time data was performed using Monte Carlo resampling with 1 000 000 iterations. Swarm plots were plotted using the Seaborn package in Python.

### Purification of Rrm3 constructs for biochemical analysis

N-terminal deletion constructs of RRM3 (comprising residues 187–723, 213–723 and 231–723) were PCR amplified from the full-length gene (originally from the yeast strain W303a) and cloned in pET28a at the NdeI/XhoI restriction sites, yielding an N-terminal His_6_-tag. The Rrm3 constructs were over-expressed in *E. coli* Rosetta2 (DE3) pLysS by induction with 0.5 mM IPTG and growth overnight at 16°C. The cells were suspended in Buffer L (50 mM Tris–HCl pH 8.0 (4°C), 500 mM NaCl, 5 mM β-ME, 10% v/v glycerol, 1 mM PMSF) + 10 mM imidazole, lysed by sonication, followed by centrifugation at 15 000 rpm for 1 h in a Beckman JA-14 rotor. The supernatant was then loaded on a HisPur Ni-NTA superflow agarose (Thermo Scientific), washed with 20 mM imidazole in buffer L and eluted with 300 mM imidazole in Buffer L. The fractions containing Rrm3 were dialyzed in Buffer P (50 mM Tris–HCl pH 7.5 (4°C), 1 mM EDTA, 1 mM DTT, 20% v/v glycerol and 0.1 mM PMSF) + 400 mM NaCl. After extensive dialysis, the sample was diluted 2-fold with buffer P (to reduce the NaCl concentration to 200 mM), loaded on a High Q column (BioRad), the flow-through collected and loaded on a High S column (BioRad) and then eluted at 1 M NaCl with Buffer P. The eluted proteins were dialyzed in storage buffer (50 mM Tris–HCl pH 8, 600 mM NaCl, 1 mM EDTA, 1 mM DTT, 40% v/v glycerol) and stored at −80°C.

### ATPase and DNA unwinding assays

The DNA-dependent ATPase activity was determined spectrophotometrically using a β-Nicotinamide adenine dinucleotide reduced disodium salt hydrate (NADH, LADH, PK, PEP) enzyme-coupled assay as previously reported (20). ATPase activity was measured at 30°C in buffer 20 mM Hepes (pH 7.4), 20 mM NaCl, 2 mM Mg-acetate and 1 mM DTT at a constant concentration of 20 nM Rrm3, 1 mM ATP, and in the presence or absence of 200 nM ssDNA (dT_60_). Fluorescence experiments were performed with an L-format PC1 spectrofluorimeter (ISS, Champaign, IL). DNA unwinding experiments were performed using a DNA substrate consisting of a double-stranded region of 20 bp and a 5′ssDNA tail of 20 nt (dT20). At the blunt-end of the DNA, the 3′-end was labeled with FAM (carboxy fluorescein) while the opposite 5′ end was labeled with the quencher Iowa Black FQ (IDT). Rrm3-dependent DNA unwinding of the 20 bp dsDNA region results in the de-queching of FAM fluorescence (λ_ex_ = 490 nm, λ_em_ = 530 nm) due to the separation of the two strands. The experiments were performed in 20 mM Hepes (pH 7.4), 20 mM NaCl, 2 mM Mg acetate, 2 mM DTT, 1mg/ml BSA, and 0.1% C12E10 at 30°C. A complex of 20 nM DNA was formed in the presence of a 4-fold excess concentration of Rrm3, and the reactions were started by the addition of 1 mM ATP and 100 nM of ssDNA trap (same as the strand carrying the quencher) to prevent re-annealing of the dsDNA region.

## Results

### Rrm3 and Pif1 division of labor at G4 sequences

**Figure 1.**
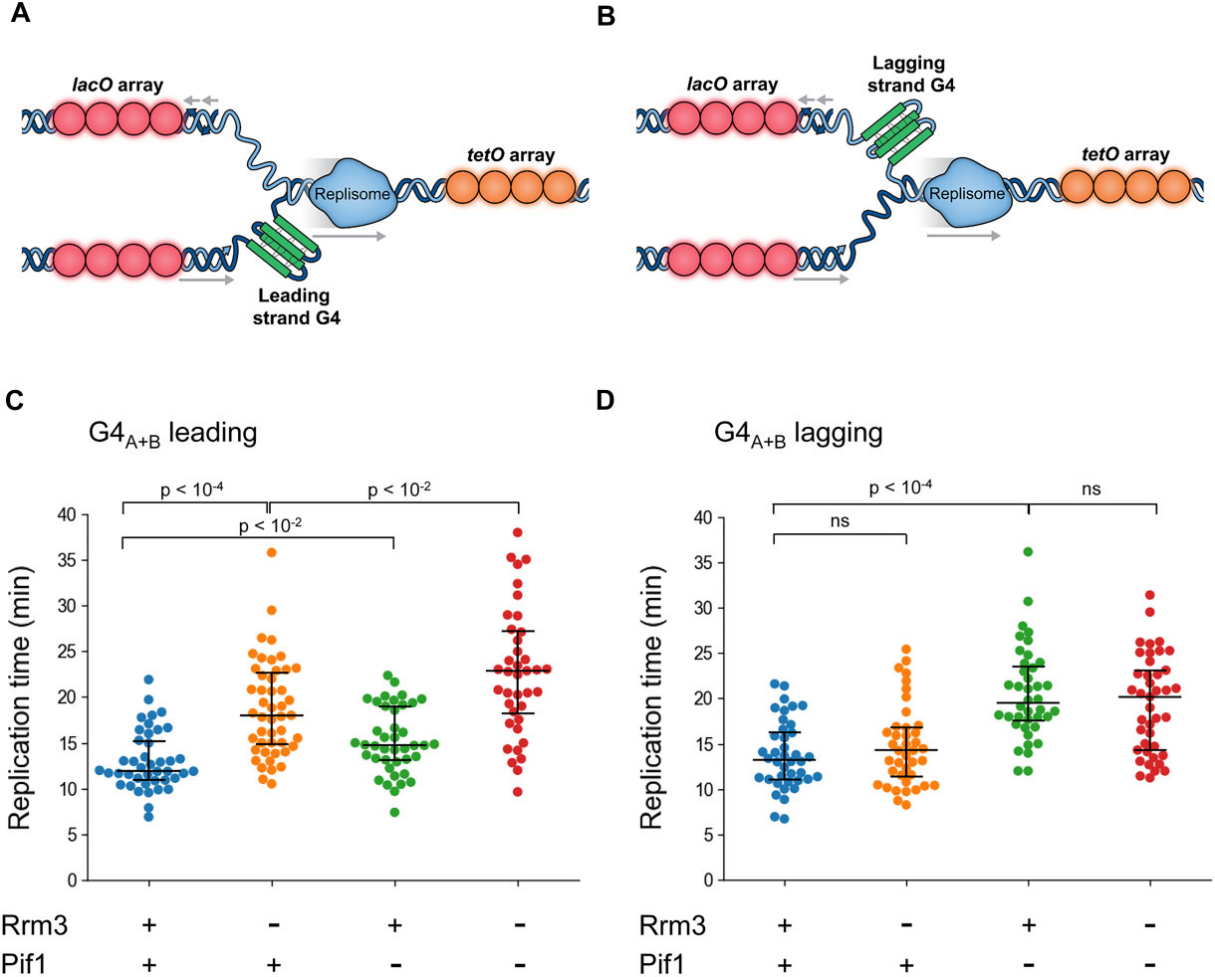
Division of labor between Rrm3 and Pif1 during replication through leading and lagging strand G4s, respectively. (A, B) Schematic illustration of the experimental system, which allows monitoring the progression of single replication forks through G4s located on the leading (**A**) or lagging (**B**) strand templates using fluorescently labeled *lacO* (dark red) and *tetO* (orange) arrays. (C, D) Replication times through a region of ∼30 kb (mid-array distance) containing G4_(A+B)_ located on the leading (**C**) or lagging strand (**D**) templates for Rrm3 expressing cells, cells depleted in Rrm3, cells containing *pif1*-deletion and cells lacking both helicases. All strains contain *RRM3*-AID, and Rrm3 depletion was induced by the addition of 1 mM IAA, as described in the Materials and methods section.

We recently described an approach for measuring the rate of DNA replication at a specific genomic locus in single live *S. cerevisiae* cells. This approach is based on monitoring the intensity of fluorescently labeled *lacO* and *tetO* arrays visualized as fluorescent dots under time-lapse microscopy (16). Tracking the intensity of each fluorescent dot in individual cells allows us to identify when each locus is being replicated and to measure progression of a single replisome through the genomic region between the arrays. We applied this approach to measure replisome progression through G4 sequences integrated between fluorescently labeled *lacO* and *tetO* arrays located downstream of *ARS413* (10). Using this approach, we previously examined replication through two tandem G4 sequences (G4_(A+B)_), derived from the yeast genome (21), located on the lagging or leading strand template (10) (Figure 1A, B).

Here, we measured replication fork progression in yeast strains containing Rrm3 fused to an auxin-inducible degron (AID), *RRM3*-AID. Rrm3 degradation in synchronized yeast populations was initiated by the addition of auxin during G1, prior to release into S phase and the initiation of DNA replication. It was previously shown, using sequencing approaches and 2D gels, that the deletion of Rrm3 leads to a significant fork stalling at tRNA genes, which are widely spread in the yeast genome (22–24). A recent study utilized a Nanopore sequencing approach for the detection of fork stalling at a head-on (HO) Glutamate tRNA gene (YNCI0011W) in *rrm3*-deletion strain (24). To examine whether our live cell microscopy system is consistent with previous measurements of fork stalling in the absence of Rrm3, we integrated this tRNA gene in a HO orientation, including upstream and downstream regions, between the *lacO* and *tetO* arrays in a *RRM3*-AID strain. Replication measurements of this strain revealed a significant replication slowdown through the tRNA upon Rrm3 depletion (Supplementary Figure S1). These results are in accordance with previous measurements using site specific or genomic approaches (22–24) highlighting the sensitivity of our approach for the detection of fork slowdown at a known roadblock due to Rrm3 depletion.

Next, we examined replication through G4s in the presence or absence of Rrm3 using the live-cell microscopy system. We found that in cells depleted of Rrm3, replication fork progression through G4_(A+B)_ placed in the leading strand template is significantly slowed down, relative to cells expressing Rrm3 (Figure 1C and Supplementary Figure S2). In contrast, Rrm3 depletion does not lead to replication slowdown through G4_(A+B)_ placed in the lagging strand template (Figure 1D and Supplementary Figure S2). These results highlight the importance of Rrm3 for enabling normal replication progression through leading strand G4s only.

Previously, we found that Pif1 is essential for efficient replication through lagging strand G4s, but not leading strand G4s (10). To further study the interplay between Pif1 and Rrm3 for enabling efficient replication through G4s, we examined replication in *pif1*Δ strains in the presence or absence of Rrm3. We found that *pif1*-deletion, generated on the background of *RRM3-AID* strain, leads to a mild slowdown of replication through leading strand G4s (Figure 1C) in the absence of IAA, but no slowdown was observed when *pif1*-deletion was generated on *RRM3* background (Supplementary Figure S3). Importantly, we found that in *pif1*Δ cells depleted of Rrm3, replication through leading strand G4_(A+B)_ is significantly slower relative to *PIF1* WT cells depleted of Rrm3 (Figure 1C). In accordance with our previous study (10), we found that in *pif1*Δ cells replication is slowed down through lagging strand G4_(A+B)_ (Figure 1D). However, further depletion of Rrm3 in the *pif1*Δ cells does not aggravate replication slowdown (Figure 1D).

To further support our findings, we utilized the copy number variation approach to examine *RRM3*-AID and *pif1*-deletion strains containing leading G4_(A+B)_. Cells were synchronized and released with or without Rrm3 depletion and samples were collected for gDNA extraction. Specifically, we collected samples during G1 phase as a control and samples following 35, 47 and 60 min after release into S-phase (Supplementary Figure S4). We performed our experiments in the presence of 50 mM hydroxyurea (HU) which slowdown fork progression by ∼6-fold (25) and thus increase the time window for the detection of copy number variations. To examine fork progression from *ARS413* through leading G4_(A+B)_, we have used qPCR with primers annealing to genomic regions located before or after the G4_(A+B)_ sequence. This gDNA analysis revealed a decrease of copy number level downstream of the G4_(A+B)_ sequence upon Rrm3 depletion, relative to Rrm3 expressing conditions (Supplementary Figure S5). Analysis of copy number upstream and downstream of *ARS607* in the presence or absence of Rrm3 reveals mild variation between the conditions (Supplementary Figure S5) that could results from natural G4 forming sequences located at the vicinity of this origin (21).

Overall, the live cell microscopy data and copy number variation analysis demonstrate the importance of Rrm3 for replication through leading G4s and the division of labor between Rrm3 and Pif1 for replication through leading and lagging G4s, respectively (Figure 1 and Supplementary Figure S5). In addition, our results reveal that in the absence of Rrm3 activity, Pif1 can partially suppress replication slowdown at leading G4s. However, in the absence of Pif1, Rrm3 cannot suppress replication slowdown at lagging G4s, highlighting the higher specificity of Rrm3 for leading G4s (Figure 1C, D).

### Replication slowdown in the absence of Rrm3 is dependent on G4 stability

**Figure 2.**
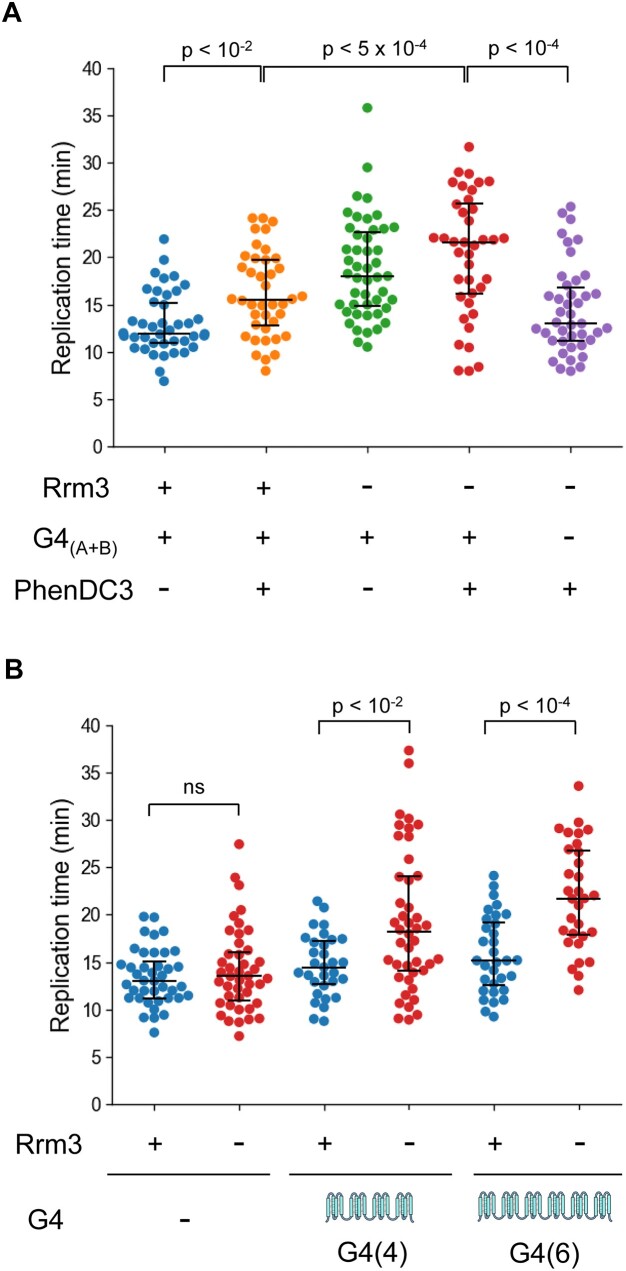
Replication fork progression through leading strand G4s in Rrm3 depleted cells is dependent on G4 stability and the number of G4 motifs. (**A**) Replication times through leading G4_(A+B)_ in the presence or absence of PhenDC3 and in the presence or absence of Rrm3. Data for strains measured in the absence of PhenDC3 is adopted from Figure 1C and is shown for comparison. (**B**) Replication times through 4 or 6 repeats (G4(4) and G4(6), respectively) of leading strand G4 sequence (G_3_T_3_)_3_G_3_, or in the absence of G4, in Rrm3 expressing (blue) or Rrm3 depleted strains (red). Depletion of Rrm3 (red) results in significant slowdowns of replication through leading G4(4) or G4(6), relative to the respective strains expressing Rrm3 (red). All strains contain *RRM3*-AID, and Rrm3 depletion was induced by the addition of 1mM IAA, as described in the Materials and methods section.

To examine the effect of leading strand G4 stability on replication fork progression in the presence or absence of Rrm3, we first used the G4 stabilizing ligand PhenDC3. Many G4 ligands have been thoroughly characterized *in vitro* and their effects were tested in yeast or in mammalian cells (26–28). Specifically, PhenDC3 was shown to increase genome instability and recombination at G4 regions in yeast cells (13,29). Here, we examined whether PheDC3 can affect replication fork progression through leading G4_(A+B)_, in WT or Rrm3 depleted cells. We found that replication fork progression through leading G4_(A+B)_ is moderately affected by the presence of PhenDC3 in cells expressing Rrm3 (Figure 2A). However, we found that replication fork progression through the leading G4s is significantly slowed down by the presence of PhenDC3 in cells depleted of Rrm3, relative to cells expressing Rrm3 (Figure 2A). To verify that PhenDC3 leads to replication slowdown strictly due to the stabilization of G4s, we examined whether PhenDC3 slows replication in the absence of G4_(A+B)_ and found no effect (Figure 2A).

To further examine whether replication fork progression through leading G4s is affected by the nature and number of the G4 motifs, we integrated different G4 repeats into *RRM3-AID* cells. Previous bioinformatics analysis of G4 sequences in the yeast genome indicated that the average number of conserved tandem G4 repeats is 5.1 (21). Thus, we chose to integrate 4 or 6 tandem repeats of a synthetic (G_3_T_3_)_3_G_3_ sequence into the leading strand template (Figure 2B, G4(4) and G4(6), respectively), in the background of a *RRM3-AID* strain. This G4 sequence was previously shown to have a moderate stability of 58.8°C (18). We found that replication progression from *ARS413* through genomic DNA that do not contain the G4s is normal in the absence of Rrm3 (Figure 2B). In contrast, we found a significant replication slowdown through G4(4) and G4(6) in cells depleted of Rrm3, relative to the Rrm3 expressing cells. To further support replication slowdown through G4(6) upon depletion of Rrm3 and examine the effect of PhenDC3, we have utilized the copy number variation approach described above. We constructed a leading G4(6) strain, integrated 3 kb from *ARS413*, containing *RRM3*-AID. Copy number variation experiments for the analysis of fork progression through G4(6) in the absence of PhenDC3 revealed a mild decrease upon Rrm3 depletion (Supplementary Figure S6). We found that the addition of PhenDC3 to Rrm3 depleted cells results in a larger decrease of copy number downstream of the G4(6), relative to Rrm3 expressing cells treated with PhenDC3 (Supplementary Figure S6). These results are in agreement with our live-cell microscopy findings demonstrating that Rrm3 is required for replication fork progression through different G4-forming sequences, and that the extent of slowdown is dependent on G4 stability.

### The importance of Rrm3 functional domains for replication through leading strand G4s

**Figure 3.**
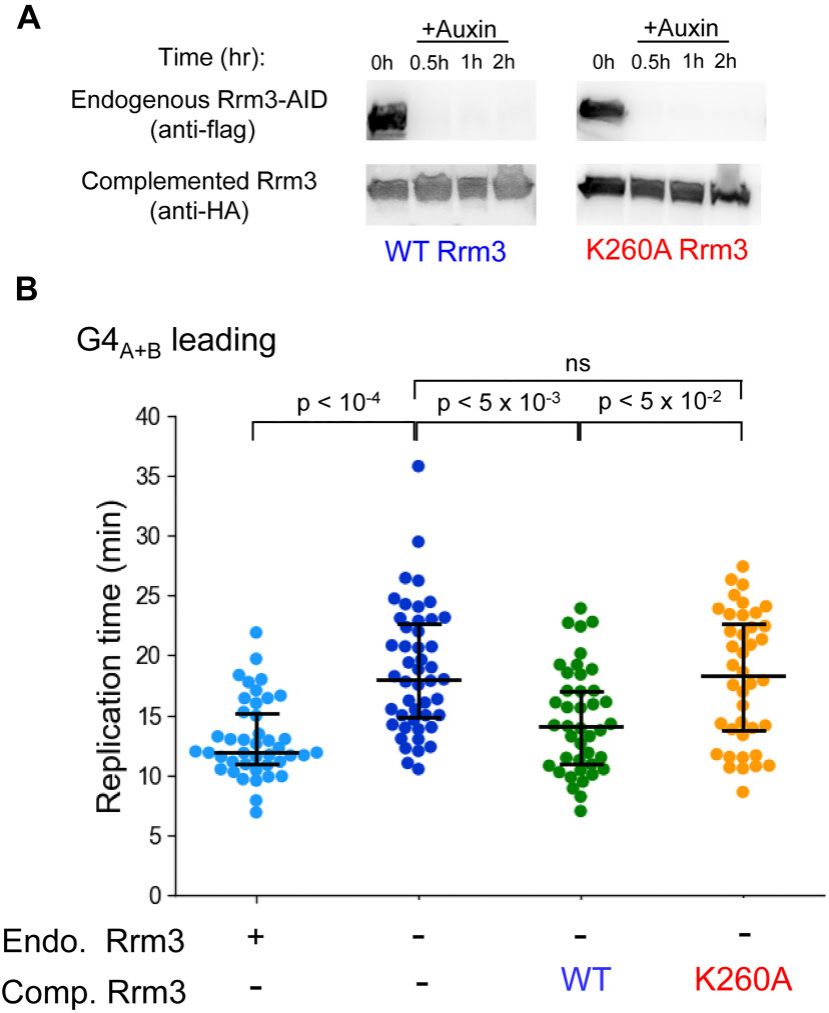
Replication through leading G4s is dependent on Rrm3 catalytic activity. (**A**) Western blot analysis for the detection of Rrm3 depletion following the addition of auxin and the complementation of cells with WT Rrm3 or the K260A mutant expressed from the native *RRM3* promoter and located at the *URA3* locus. (**B**) Replication times through leading G4_A+B_ of cells expressing the endogenous Rrm3 (WT), cells depleted of Rrm3, and cells complemented with WT Rrm3 or the K260A mutant. Data for replication through leading G4_A+B_ in the presence or absence of endogenous Rrm3 is adapted from Figure 1C and is shown for comparison. All strains contain endogenous *RRM3*-AID, and endogenous Rrm3 depletion was induced by the addition of 1 mM IAA, as described in the Materials and methods section.

To examine the importance of Rrm3 helicase activity for replication through leading strand G4 sequences, we examined replication in strains expressing the K260A Rrm3 ATPase dead mutant (30). To enable the complementation of Rrm3 WT and mutant in Rrm3 depleted cells, we integrated a second copy of *RRM3* into the *URA3* locus on the background of the *RRM3-AID* strain. Using these strains, we first verified that the endogenous Rrm3 is depleted upon auxin addition and that the complemented Rrm3 WT and K260A mutant are expressed to a similar level (Figure 3A). Next, we performed live cell microscopy experiments to measure replication fork progression through the leading strand G4_(A+B)_ in the complemented WT or K260A cells. We found that while the complementation with WT Rrm3 restores normal replication through G4_(A+B)_, replication in the K260A cells is significantly slowed down, similar to Rrm3 depletion (Figure 3B). This indicates that the helicase activity of Rrm3 is essential for efficient replication fork progression through leading strand G4 sequences.

Previous studies revealed that Rrm3 contains a non-conserved intrinsically disordered N‐terminal domain and a conserved helicase domain (9). To map the borders of the Rrm3 N-terminal unstructured domain and the helicase domain, we performed AlphaFold structural prediction of Rrm3 (31) and IUPred sequence analysis (32). Using these tools, we verified that the N-terminal unstructured domain of Rrm3 spans residues 1–230, followed by a well-conserved and folded helicase domain (9) (Supplementary Figure S7). A previous study has identified a PCNA interacting protein (PIP) motif in the N-terminal domain, located between residues 35–42, and showed that deletion of the first 54 residues of Rrm3 (Δ54) completely abolishes its interaction with PCNA (33). In addition, a recent study identified the importance of residues 186–212 for controlling DNA replication under hydroxyurea replication stress and as a site of interaction with Orc5 unrelated to Rrm3 helicase activity (34).

**Figure 4.**
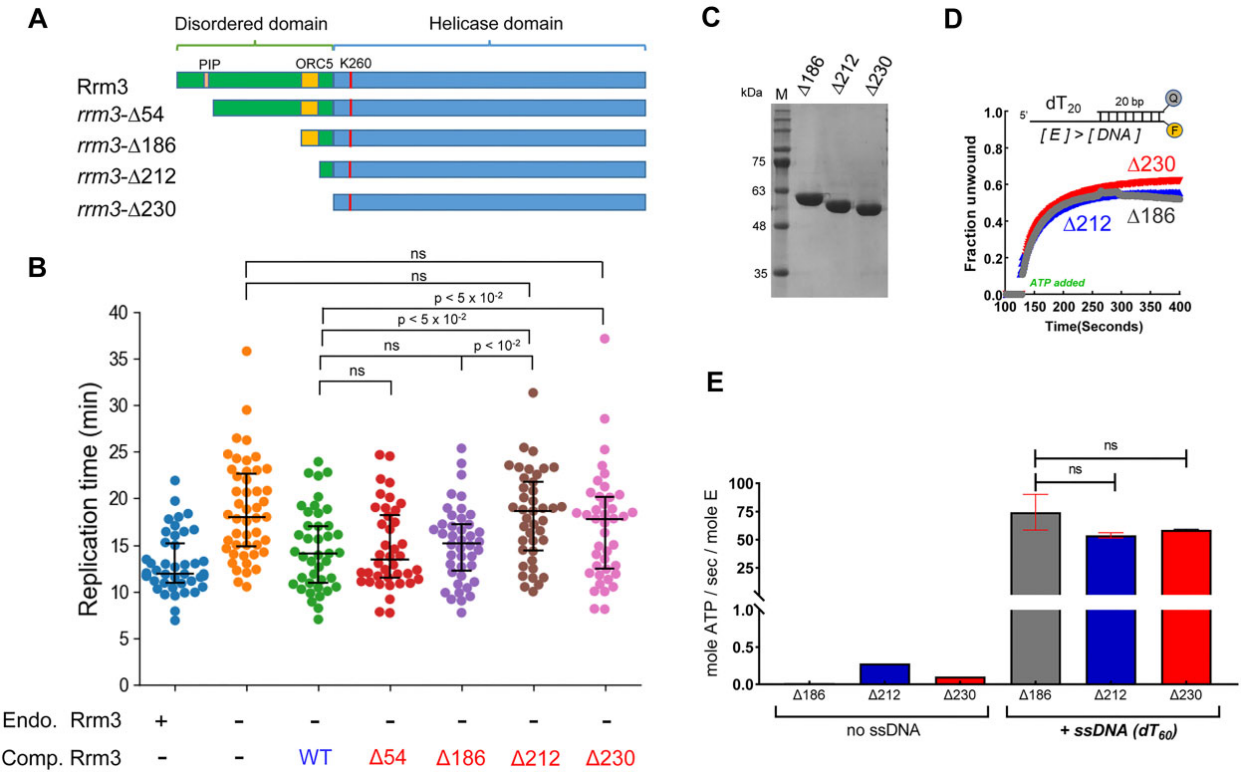
Fast replication through leading strand G4s requires Rrm3 N-terminal domain. (**A**) Schematic illustration of the different truncated Rrm3 variants examined including *rrm3*-Δ54 for the deletion of the PIP region in Rrm3, *rrm3*-Δ186, *rrm3*-Δ212 and *rrm3*-Δ230 for the deletion of the whole N-terminal domain. (**B**) Replication times through leading G4_A+B_ of WT cells, cells depleted in Rrm3, cells complemented with WT or different N-terminal truncated Rrm3 shown in (A). Truncation of the first 212 or 230 residues leads to a dramatic slowdown in replication relative to full length Rrm3, highlighting the importance of these residues for replication through leading G4s. Data for replication through leading G4_A+B_ in the presence or absence of endogenous Rrm3 is adapted from Figure 1A and is shown for comparison. (**C**) Coomassie stained SDS-PAGE showing the recombinantly purified *rrm3*-Δ186, *rrm3*-Δ212 and *rrm3*-Δ230 variants. (D, E) Helicase (**D**) and ATPase (**E**) activity of the different truncated Rrm3 versions in (C) showing no significant difference in activity between the different truncations.

To examine the importance of the different functional regions of Rrm3 N-terminal domain for replication through leading G4s, we constructed a series of truncated versions of Rrm3 consisting of *rrm3*-Δ54, *rrm3*-Δ186, *rrm3*-Δ212 and *rrm3*-Δ230 (Figure 4A). We introduced these truncated versions of Rrm3 into the *RRM3-AID* strain and verified their expression by western blot analysis (Supplementary Figure S8). Next, we examined the effect of the different Rrm3 truncated versions on replication through leading G4_(A+B)_ in cells depleted of the endogenous Rrm3. We found that the *rrm3*-Δ54 variant facilitates normal replication through the G4s (Figure 4A), despite lower expression of this variant (Supplementary Figure S8). These results show that the interaction of Rrm3 with PCNA is not essential for fork progression through the leading strand G4_(A+B)_ and that low expression of Rrm3 is sufficient for efficient replication at this G4 sequence. Moreover, we found that the *rrm3*-Δ186 variant, lacking 186 aa from the N-terminus, also facilitates replication through G4_(A+B)_ at similar rates (Figure 4B). In contrast, both the *rrm3*-Δ212 and *rrm3*-Δ230 variants displayed a significant slowdown in leading strand G4 replication. This slowdown is similar to the slowdown observed in the Rrm3 depleted strain (Figure 4B). To verify that the slowdown in replication of the *rrm3*-Δ212 and *rrm3*-Δ230 variants is not due to reduced enzymatic activities, we performed biochemical assays to characterize the purified truncated Rrm3 variants (Figure 4C). Theses assays revealed that *rrm3*-Δ186, *rrm3*-Δ212 and *rrm3*-Δ230 variants are all active and have similar ATPase and DNA unwinding activities (Figure 4D,E). Overall, these results show that the N-terminal domain is essential for replication fork progression through leading strand G4s. However, while all large N-terminal truncation variants have similar enzymatic activities *in vitro*, truncations larger than Δ212 lead to a slowdown of replication through leading strand G4s *in vivo*. These observations suggest a yet uncharacterized role of the region spanning amino acids 186 to 212 in regulating Rrm3 function.

## Discussion

Here, we discovered that Rrm3 is essential for proper replisome progression through different G4 sequences located on the leading strand template (Figures 1 and 2). The importance of Rrm3 catalytic activity for leading G4 replication (Figure 3) suggests that either Rrm3 helicase activity is needed to unwind G4 structures or its sweepase activity (15,35) is used to remove proteins bound to them. In either case, enzymatically active Rrm3 is required for preventing fork stalling during leading strand replication at G4 forming sequences. Previous studies revealed the importance of Rrm3 in preventing replisome stalling at more than a thousand genomic sites, including centromeres, telomeres, tRNA genes and ribosomal DNA (15). Fork progression through these sites was shown to be dependent on Rrm3 catalytic activity as well (6). Since these pausing sites overlap with sites of stable protein-DNA interactions, it was suggested that Rrm3 could remove protein–DNA complexes ahead of the replication fork (15). However, many of these sites, including gene promoters and telomeres, also contain G4 motifs, raising the possibility that Rrm3 activity at G4s can contribute to replisome progression at these regions. Our findings that Rrm3 facilitates fork progression through leading strand G4s support this possibility, expanding the different functions of Rrm3 during genome replication.

Our current findings that Rrm3 is essential for normal replication only through leading strand G4s (Figure 1) complement our results showing that Pif1 is essential for normal replication through lagging strand G4s (10). These findings highlight a clear division of labor between Rrm3 and Pif1 in enabling efficient replisome progression through leading and lagging strand G4 sequences, respectively. Previous studies characterized the number, distribution, and conservation of G4 DNA motifs in yeast (2,21). Based on sequence pattern criteria and conservation, more than 650 G4 sequences were identified in the yeast *Saccharomyces cerevisiae* distributed over all 16 yeast chromosomes (21). Since the yeast genome contain hundreds of G4s and origins of replication (21,36,37), the specialized activities of Rrm3 and Pif1 may enable high flexibility of origin firing for efficient fork progression through G4 sequences located on the leading or lagging strand templates. Such flexibility in G4 replication directionality can be particularly useful when origin firing program is altered due to replication stress and checkpoint activation (38). While we observed a division of labor between Rrm3 and Pif1, our findings that Pif1 can reduce the level of replication slowdown through leading G4s in the absence of Rrm3, reveal some level of cooperation between the two helicases (Figure 1). Interestingly, Rrm3 cannot act on lagging G4s in the absence of Pif1, highlighting its higher specificity in promoting leading strand G4s replication. Cooperation between Rrm3 and Pif1 has been previously observed for replication fork progression through different genomic regions, including ribosomal DNA, tRNA genes and centromeres (7,24,30).

At the molecular level, the ability of Rrm3 to facilitate fork progression through leading strand G4s can be explained by previous findings showing that Rrm3 physically interacts with Pol2, the catalytic subunit of the leading strand-specific DNA polymerase ϵ in yeast (39). This interaction suggests that Rrm3 activity at leading strand G4s is tightly coupled to polymerase progression on the leading strand template. Our results showing the importance of Rrm3 N-terminal region for replication through leading G4s, specifically residues 186–212 (Figure 4), suggest that this region may be important for Rrm3-Pol2 interaction; however, additional experiments are needed to test this possibility. Interestingly, while Rrm3 contains, within the first 54 amino acids, a PIP box that enables its interaction with PCNA (33), deletion of this N-terminal region did not affect fork progression through leading strand G4 sequences (Figure 4). In agreement with these findings, previous studies showed that deletion of Rrm3 PIP region had no effect on Rrm3 control of DNA synthesis during replication stress (34). In contrast, it was shown that mutation or deletion of two PIP regions in Pif1 reduces fork progression through lagging strand G4s (10) and decreases the efficiency of break induced replication (40). While PCNA is located on both leading and lagging strand replication machineries (41), the high functional importance of Pif1-PCNA interaction for replication through lagging G4s is correlated with the importance of PCNA interaction with other lagging strand proteins including Cdc9 ligase, Fen1 and Polymerase δ for lagging strand DNA synthesis (42–44).

## Supplementary Material

gkad1205_Supplemental_File

## Data Availability

All data and code for analysis are available upon request.
