# Supplementary material for: Rrm3 and Pif1 division of labor during replication through leading and lagging strand G-quadruplex
Source: Nucleic Acids Res. 2023 Dec 20;52(4):1753–62. doi: 10.1093/nar/gkad1205 (PMC10899776; doi:10.1093/nar/gkad1205)
Supplement: gkad1205_Supplemental_File [file gkad1205_supplemental_file.pdf]

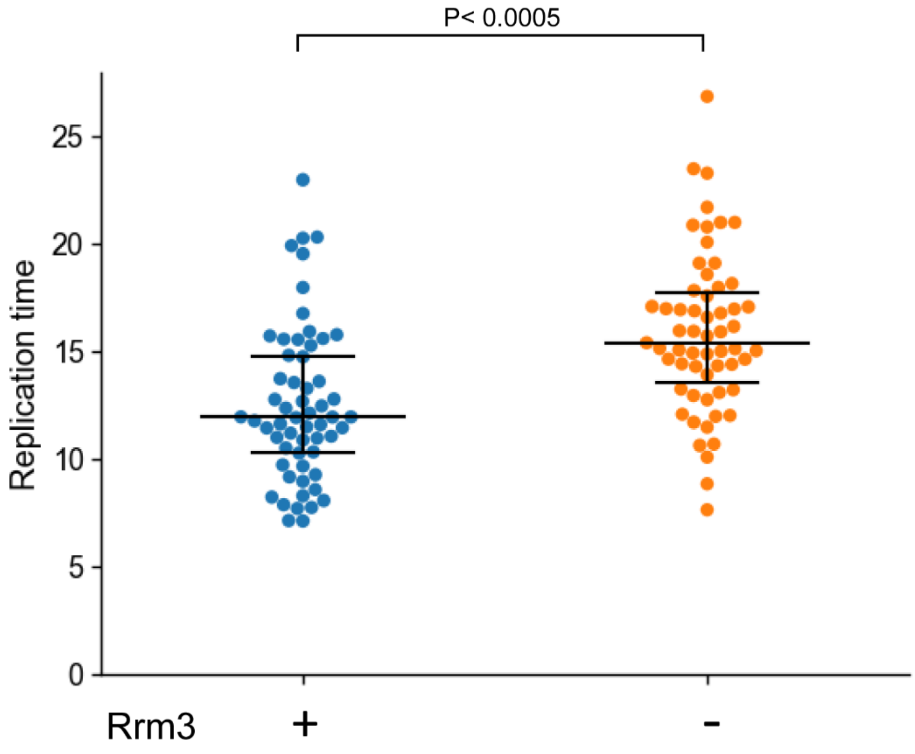

Figure S1: Replication times through Glutamate tRNA (YNCI0011W) located between the *lacO* and *tetO* arrays in a head-on (HO) orientation, measured for cells expressing Rrm3 (blue) or for cells depleted of Rrm3 (orange). Replication through the tRNA-Glu is significantly slowed down upon Rrm3 depletion in agreement with previous studies showing replication stalling at tRNAs in *rrm3*-deleted strains (see text for details). The Glutamate tRNA strain contains *RRM3*-AID and Rrm3 depletion was induced by the addition of 1mM IAA, as described in the Materials and Methods section.

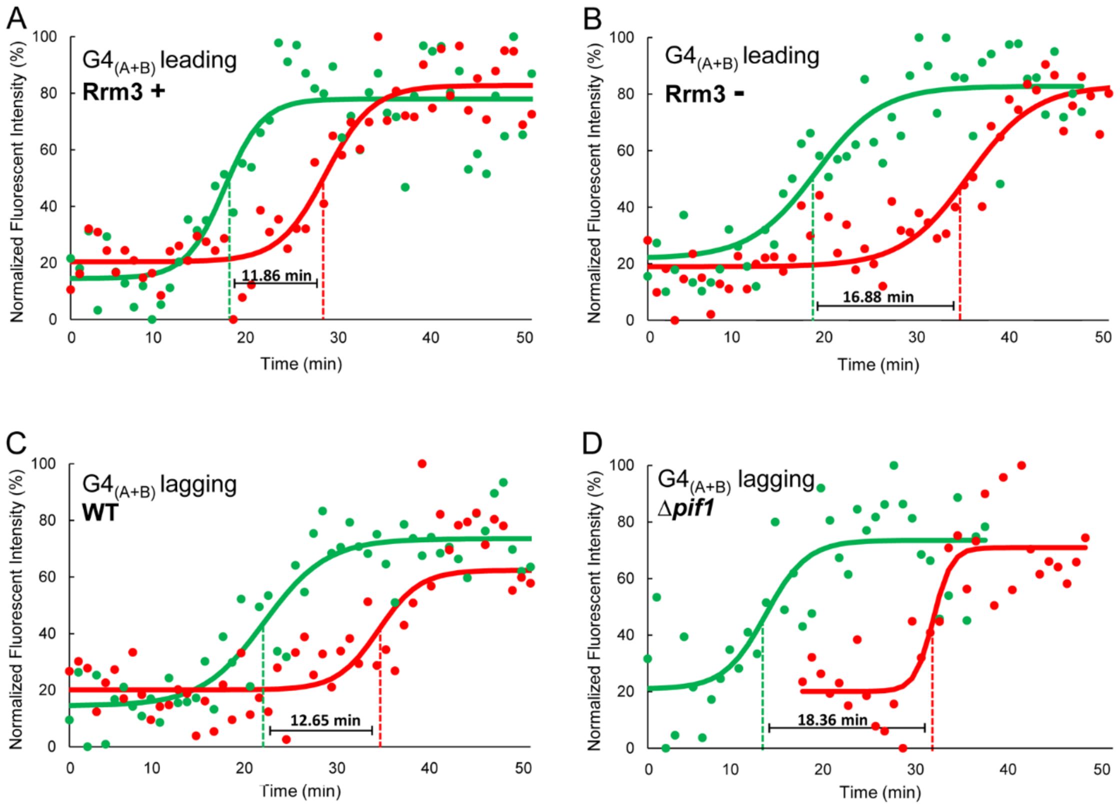

Figure S2: Representative results of single cell analysis showing replication times of ~30 Kb (distance between mid-*lacO* and mid-*tetO* arrays) in cells containing $G4_{(A+B)}$ between the arrays. (**A-B**) Cells from WT strain containing leading $G4_{(A+B)}$ in the presence of Rrm3 (**A**) or in the absence of Rrm3 (**B**). (**C-D**) representative cells from WT (**C**) and *pif1*-deletion (**D**) strains containing lagging $G4_{(A+B)}$. Solid lines represent a fit of the data to a sigmoidal function, green and red mid-points are indicated by dashed lines. Replication time for each cell is shown as the difference in midpoints of the sigmoidal fit between the red (*tetO*) and green (*lacO*) channels. Strains of panels **A-B** contain *RRM3*-AID, and Rrm3 depletion was induced by the addition of 1mM IAA, as described in the Materials and Methods section.

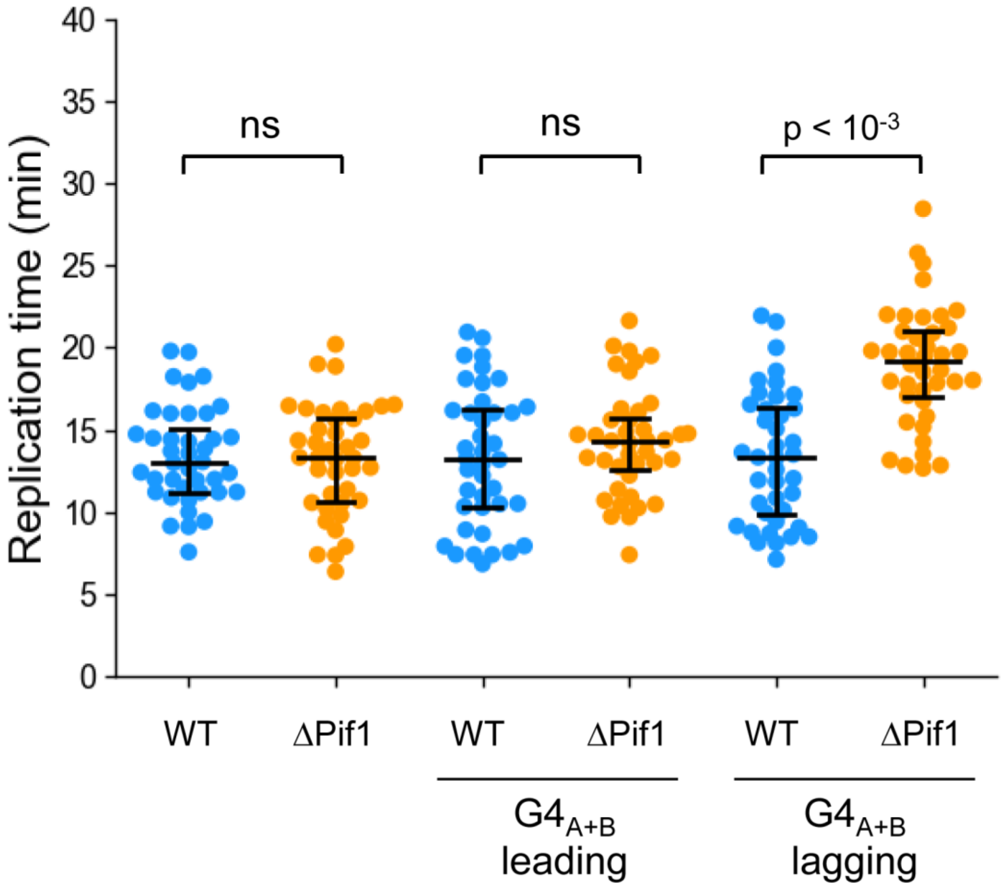

Figure S3: Replication times through a region of ~30 kb containing $G4_{(A+B)}$ located on the leading or lagging strand templates, measured for WT (blue) or *pif1*-deletion strains (orange). Replication times in the absence of G4s for the two strains is shown for comparison. Longer replication times through lagging $G4_{(A+B)}$ in *pif1*-deletion strain, relative to WT strain, indicate slower fork progression through the G4 sequence. No significant replication slowdown is observed for replication through leading $G4_{(A+B)}$ in the *pif1*-deletion strain, relative to WT strain.

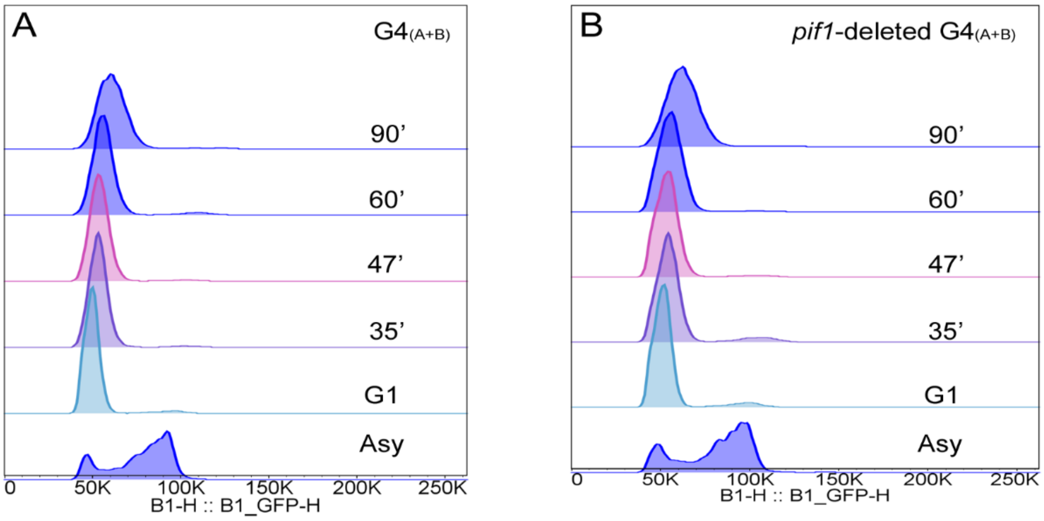

Fig. S4: Synchronization and release of strains containing *RRM3-AID* (**A**) or *RRM3-AID* and *pif1*-deletion strain (**B**) and leading $G4_{(A+B)}$ located 3 kb from *ARS413*. Samples for flow cytometry analysis were taken from asynchronized population (Asy) following 2:30 h of synchronization with alpha factor (G1) and at different time points (30, 47, 60 and 90 min) following release into S phase.

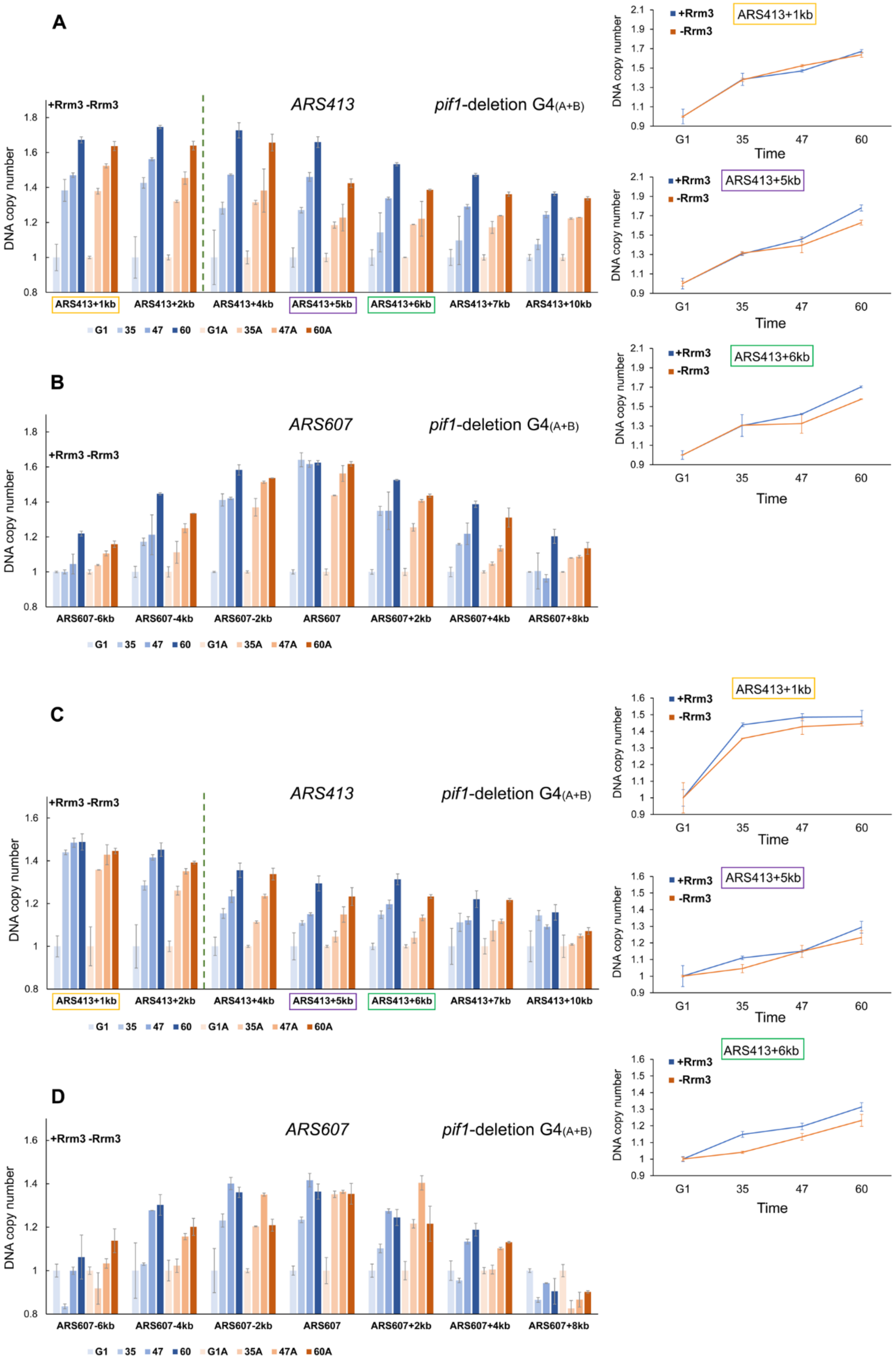

Fig. S5: Copy number variation experiments in *RRM3*-AID and *pif1*-deletion strain containing leading $G4_{(A+B)}$ following cell release into S-phase, indicating a decrease in copy number downstream of $G4_{(A+B)}$

(located 3 kb from *ARS413*, represented by green line) upon Rrm3 depletion. (**A-D**) Blue color and brown color bars are copy number levels of gDNA extracted from G1 cells, cells following 35 min, 47 min and 60 min of release, in the presence of Rrm3 (+Rrm3) and in the absence of Rrm3 (-Rrm3), respectively. Normalization was performed relative to G1 levels. Panels **A** and **C** show qPCR results for primers located at the vicinity of *ARS413* and green line represent the location of $G4_{(A+B)}$, 3 kb from *ARS413*. Right of panels **A** and **C** are kinetics of duplication of *ARS413*+1kb (orange box, top), *ARS413*+5kb (purple box, middle) and *ARS413*+6kb (green box, bottom) monitored by DNA copy number variation showing less efficient replication of regions downstream of the $G4_{(A+B)}$. Panels **B** and **D** show qPCR results for primers located downstream or upstream of *ARS607.* This analysis reveals mild variation between Rrm3 expressing and Rrm3 depleted samples that could result from natural G4 forming sequences located at the vicinity of *ARS607* (see ref 20 main text). Results for each primer pair represent an average of two independent technical repeats and error bars represent the standard error of the mean (SEM). Panels **A** and **B** and panels **C** and **D** are two independent biological repeats of the experiment.

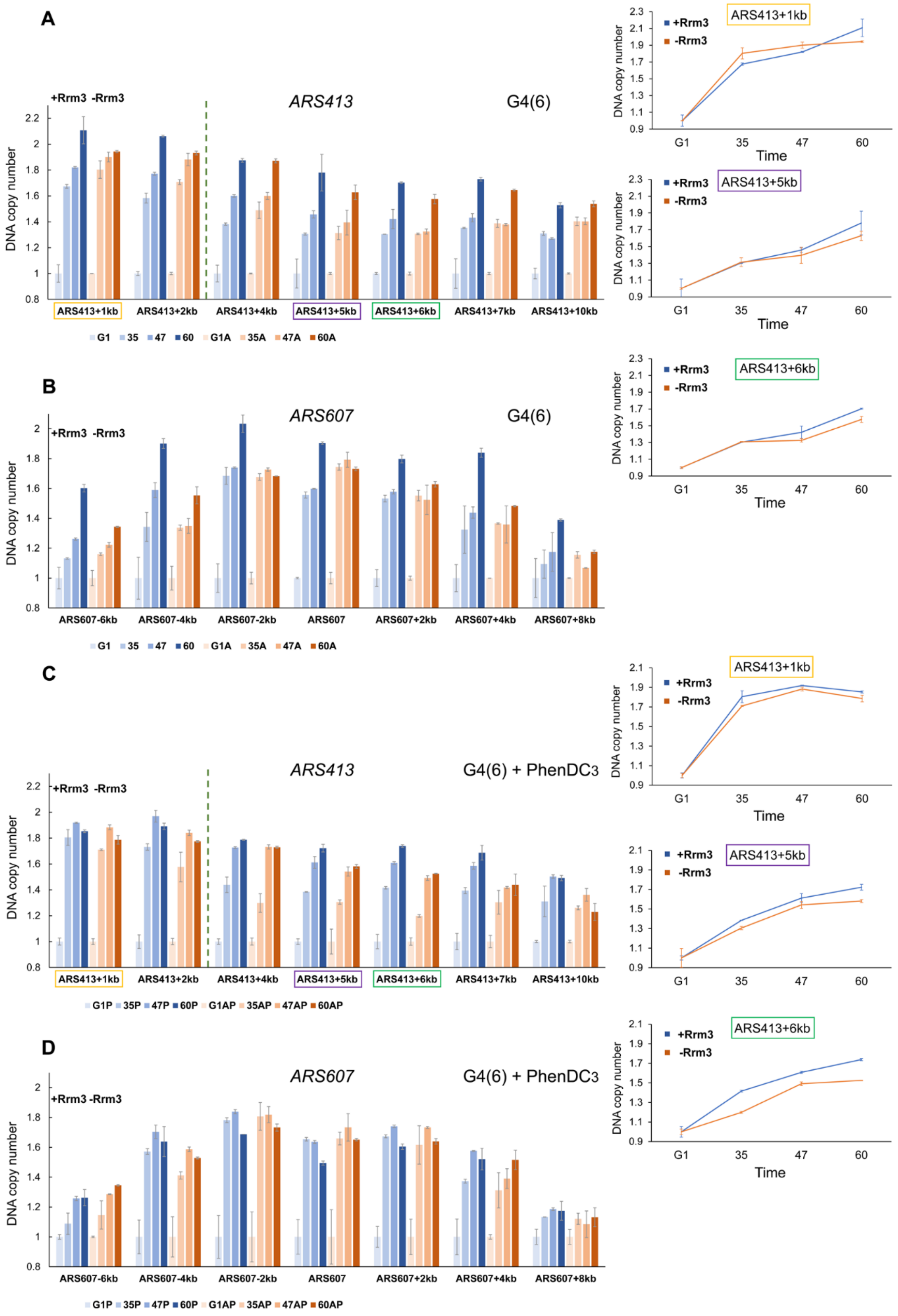

Fig. S6: Copy number variation experiments in *RRM3*-AID strain containing leading G4(6) following cell release into S-phase in the absence or presence of PhenDC3 ligand, indicating a decrease in copy

number downstream of G4(6) (located 3 kb from *ARS413*, represented by green line) upon Rrm3 depletion. (**A-D**) Blue color and brown color bars are copy number levels of gDNA extracted from G1 cells, cells following 35 min, 47 min and 60 min of release in the presence of Rrm3 (+Rrm3) and in the absence of Rrm3 (-Rrm3), respectively. Normalization was performed relative to G1 levels. Panels **A-B** and **C-D** are for experiments performed in the *absence* of PhenDC3 and in the *presence* of 10 μM of PhenDC3, respectively. Panels **A** and **C** show qPCR results for primers located at the vicinity of *ARS413* and green line represent the location of G4(6), 3 kb from *ARS413*. Right of panels **A** and **C** are kinetics of duplication of *ARS413*+1kb (orange box, top), *ARS413*+5kb (purple box, middle) and *ARS413*+6kb (green box, bottom) monitored by DNA copy number variation showing less efficient replication of regions downstream of the G4(6). Panels **B** and **D** show qPCR results for primers located downstream or upstream of *ARS607.* This analysis reveals mild variation between the conditions that could result from natural G4 forming sequences located at the vicinity of *ARS607* (see ref 20 main text). Results for each primer pair represent an average of two independent technical repeats and error bars represent the standard error of the mean (SEM).

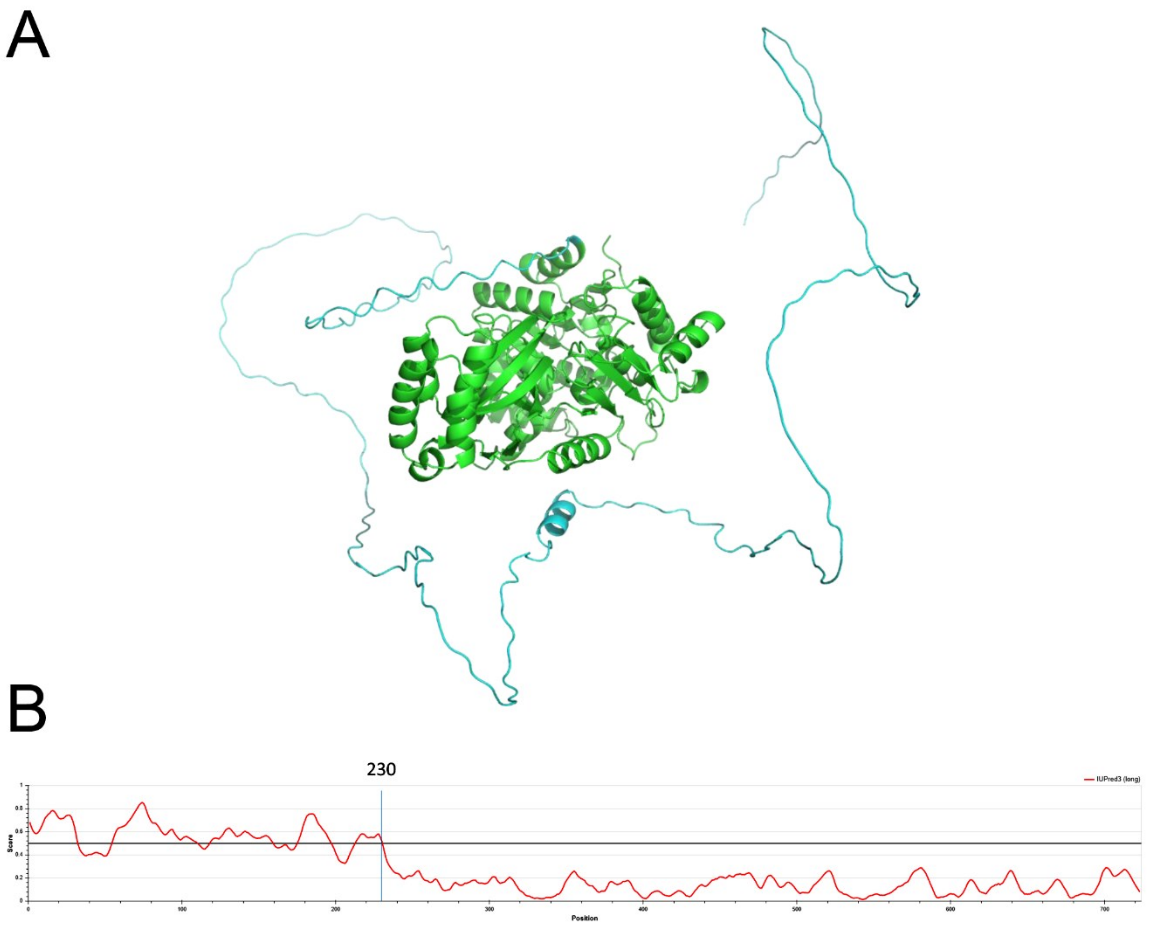

Figure S7: (**A**) A structural model of Rrm3 generated by the alpha-fold server (https://alphafold.ebi.ac.uk/). The N-terminal region of Rrm3 composed of residues 1-230 is unstructured (cyan). In contrast, residues 230-723 of Rrm3 are well structured containing α-helical and β-strands forming the helicase domain (green). (**B**) Prediction of intrinsically disordered regions in Rrm3 using the IUPred3 algorithm. The score of prediction (0-1) is plotted against Rrm3 residue number. The threshold of intrinsically disordered region detection is 0.5 and residues with a higher score are considered to be disordered. In accordance with the alpha-fold Rrm3 structural prediction (**A**) residue 1-230 are predicted to be disordered.

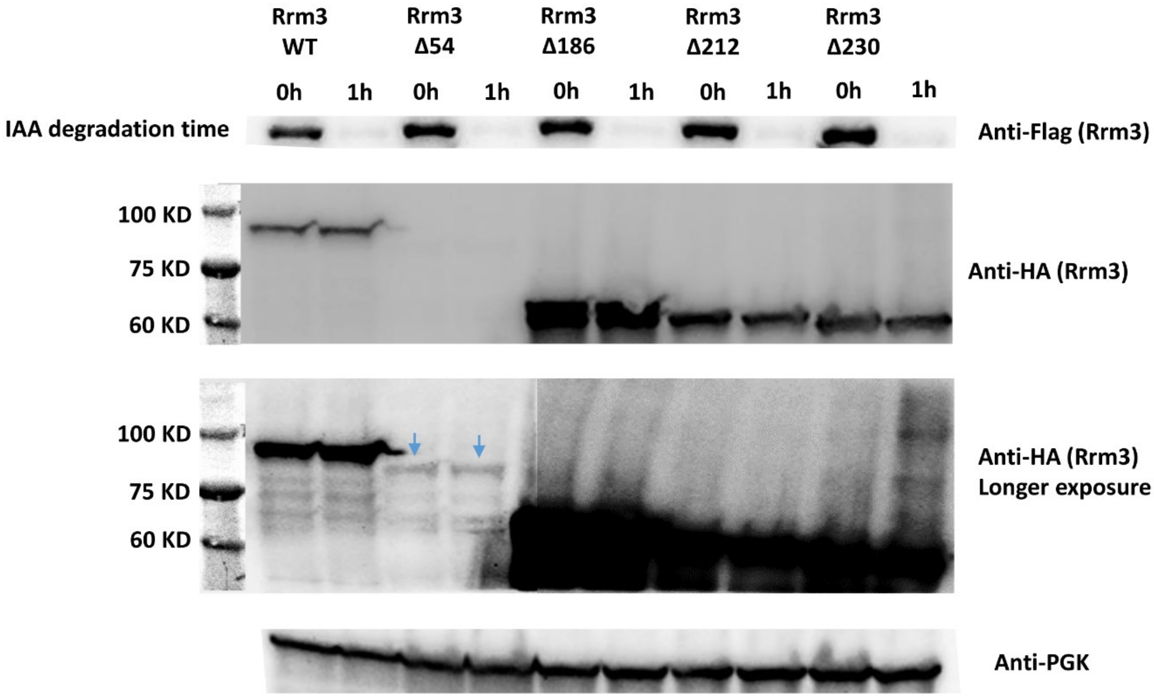

Figure S8: Western blot analysis for the detection of Rrm3-AID depletion following auxin (IAA) addition and the complementation of cells with WT Rrm3 and different N-terminal truncated variants of Rrm3 expressed from the native *RRM3* promoter. The different N-terminal truncated Rrm3 variants include *rrm3*-Δ54, *rrm3*-Δ186, *rrm3*-Δ212 and *rrm3*-Δ230. Detection of Rrm3-AID depletion was performed using anti-FLAG antibody just before (0 h) or 1 h following IAA (auxin) addition (1 mM of IAA) to the media (top). Detection of the complemented Rrm3-6xHA variants, expressed from the *URA3* locus, was performed using anti-HA antibodies. Longer exposure of the membrane was performed to detect the *rrm3*-Δ54 variant that is expressed at a low level (middle). Detection of Pgk1 using anti-Pgk1 antibody was performed as a loading control (bottom). For details regarding procedure and antibodies, see Materials and Methods section.

Table S1: Primers for qPCR analysis at the vicinity of *ARS413* and *ARS607*

Oligo name	Oligo sequence	Oligo length
ARS413 +1kb_F	GGAAGAGGCTCAAGCAATTC	20
ARS413 +1kb_R	CATTTTGCCACCGTGTCACT	20
ARS413 +2kb_F	GACGTTCAGTTAGTACCACC	20
ARS413 +2kb_R	TCTTTTGGGTCTACCACCAC	20
ARS413 +4kb_F	AGAAGCAAAGGGGTAGTACG	20
ARS413 +4kb_R	TAAGACAGGCAGACCTTGAG	20
ARS413 +5kb_F	TTGTGAGACAACAGACGCAC	20
ARS413 +5kb_R	CAAGGCGAGAGGTGGAAAAT	20
ARS413 +6kb_F	TGGAATCCGCCACTTTGAAC	20
ARS413 +6kb_R	TTCGTTGTTACCGCAAGCCA	20
ARS413 +7kb_F	GGATTGCTACCATTCACGGT	20
ARS413 +7kb_R	CCTCATCAACAGATGGAGCA	20
ARS413 +10kb_R	CGATTTCTGCGTCCTTGTAC	20
ARS413 +10kb_R	TAGCAACGCCCCCATTATTC	20
ARS607-6kb_F	ATGATGGTTGGAACTCGCGT	20
ARS607-6kb_R	AAACCTGCCTCTATGGAACC	20
ARS607-4kb_F	AGTTGGTGGCAACACAGAGA	20
ARS607-4kb_R	TACTGATCCAAGTCGCTAATG	21
ARS607-2kb_F	CCTTTAGTGCTATTTTGCCTG	21
ARS607-2kb_R	GATTCCATGCTATATGTCACG	21
ARS607_F	CTAGATCTGGAGTGACCAAAA	21
ARS607_R	AGTCTCAAAATTCTTTTCTTCCA	23
ARS607+2kb_F	TATTACCAGTACTCACATGCG	21
ARS607+2kb_R	GTGATCTTGTGCTGCTATTCA	21
ARS607+4kb_F	GATTTCTTTGCCACCCGTGA	20
ARS607+4kb_R	GCATCTGGAAGTTCAACATGA	21
ARS607+8kb_F	GGTTCGAAATCGAATAAAATAGT	23
ARS607+8kb_R	TGGATGGCGCGATCTTCTAT	20
